# Enhancing genomic prediction of wheat resistance to Fusarium head blight through integration of passive resistance traits

**DOI:** 10.3389/fpls.2026.1885296

**Published:** 2026-07-15

**Authors:** Andrii Gorash, Shayan Syed, Gintaras Brazauskas

**Affiliations:** 1 Department of Cereal Breeding, Institute of Agriculture, Lithuanian Research Centre for Agriculture and Forestry, Akademija, Lithuania; 2 Laboratory of Genetics and Physiology, Institute of Agriculture, Lithuanian Research Centre for Agriculture and Forestry, Akademija, Lithuania

**Keywords:** fusarium head blight, genomic prediction, GWAS, spring wheat, wheat resistance to FHB

## Abstract

Fusarium head blight (FHB) is one of the most devastating wheat diseases worldwide. Wheat resistance to FHB is controlled by multiple small-effect genes involved in active and passive resistance mechanisms. Complex resistance can be successfully managed using genomic selection. However, the efficiency of wheat genomic selection for FHB is restricted by prediction accuracy. In this study, we investigated the contribution of passive resistance traits in genomic prediction to FHB. A panel of 335 spring wheat genotypes were phenotyped for FHB severity and eight key traits associated with passive resistance in 2022, 2023, and 2025. Genome-wide association analysis (GWAS) was performed to identify MTAs significantly associated with FHB resistance and passive resistance traits. Genomic prediction was performed using Reproducing Kernel Hilbert Space regression (RKHS), Ridge Regression Best Linear Unbiased Prediction (rrBLUP), and Random Forest regression (RF) models. The contribution of passive resistance was evaluated by performing genomic prediction on separate sets of SNPs that were significantly associated with FHB resistance and morpho-phenological traits in GWAS. Genomic prediction based on set 1 from cross-validated GWAS outperformed the prediction of the entire set of markers (18,417 SNPs) in all models. Comparing the genomic prediction performance on the entire set of markers with that based on set 1 from cross-validated GWAS and fixed covariates (days to heading and anther extrusion), an absolute increase in accuracy of 0.10 (0.60 vs. 0.70), and 0.08 (0.62 vs. 0.70) was observed using the RKHS, and rrBLUP models, respectively. In RF model, DH and AE were included as additional predictors and the increase was 0.11 (0.53 vs. 0.64). The results indicate that the genomic prediction of wheat resistance to FHB can be improved integrating passive resistance traits into prediction models.

## 1 Introduction

Wheat (*Triticum aestivum* L.) is a principal staple crop, which is cultivated on about 219 million hectares with a global production of ~800 million tons, contributing approximately 20% of the daily caloric intake of humans globally (FAOSTAT, 2026). Food safety strongly depends on maintaining sustainable and increasing wheat yields to meet the food demands of a growing population. One of the main restrictive factors contributing to the gap between potential and actual wheat yields is the damage caused by pathogens and pests. Global yield losses due to disease epidemics and pest infestations range from 10 to 28% (Savary et al., 2019). Among wheat diseases, Fusarium head blight (FHB) is particularly devastating. In addition to yield reduction, FHB poses a serious threat to food and feed safety due to the accumulation of mycotoxins, such as deoxynivalenol (DON), T-2, nivalenol (NIV), and zearalenone (ZEN), which contaminate grain and make it unsafe for human and animal consumption. Recently, the extensive use of monoculture, zero-tillage practices, and host-consecutive crop rotation (wheat-maize) has raised unprecedented levels of disease pressure. Moreover, to limit the excessive use of hazardous chemicals, the European Union has suggested the “Farm to Fork” strategy to reduce the overall use and risk of chemical pesticides by 50% until 2030 (European Commission, 2023). Therefore, there is an urgent need to develop wheat cultivars with improved genetic resistance to FHB using eco-friendly and cost-effective breeding strategies (Zhang et al., 2021).

FHB resistance is typically divided into two categories: active and passive, as well as a combination of both (Buerstmayr et al., 2020). Passive resistance is associated with morphological traits that act as the first layer of defense, allowing plants to avoid infection. However, the other layers of defense (i.e., the 2nd and 3rd) are recognized as active resistance (Gorash et al., 2021). In addition, active resistance involves several physiological pathways and genetic interactions to detect and restrict pathogen spread (Gorash et al., 2021; Mesterhazy, 2024). Moreover, traits such as plant height, anther extrusion, longer spikes, distance between the internodes, heading, and flowering time are associated with passive resistance to FHB (Buerstmayr et al., 2009; Jones et al., 2018; Jones, 2015; Mesterhazy, 2024). Among these traits, plant height is a well-documented morphological trait associated with FHB resistance. Numerous researchers have observed the effects of reduced height genes (*Rht*) on increasing FHB susceptibility in wheat. Generally, taller plants demonstrate higher resistance, especially under natural disease pressure, demonstrating the significant role of plant height in avoiding FHB infection (Berraies et al., 2023; Holzapfel et al., 2008; Mesterházy, 1995; Srinivasachary et al., 2009). Additionally, anther extrusion is recognized as a notable trait that influences FHB resistance. In 1933, researchers revealed that anthers, which are not fully extruded serve as a substrate for pathogens to colonize (Pugh et al., 1933). Further studies have indicated that the degree of anther extrusion is associated with FHB resistance, as retained or partially extruded anthers can enhance the risk of FHB infection in wheat (Graham and Browne, 2009; He et al., 2016; Kubo et al., 2013; Skinnes et al., 2008; Xu et al., 2020). It has also been reported that anther extrusion is controlled by multiple QTLs, contributing to a low-to-medium effect (Buerstmayr and Buerstmayr, 2015; Muqaddasi et al., 2019; Okada et al., 2019; Skinnes et al., 2010). In addition, Mesterházy (1995) related passive resistance mechanisms to morphological traits, such as type I with plant height, type II with the presence/absence of awns, type III with spike density, and type IV associated with escape-related traits, such as heading/flowering and anther extrusion (Mesterházy, 1995; Nannuru et al., 2022). The mechanism behind active resistance comprises five components of resistance (Mesterhazy, 2024; Schroeder and Christensen, 1963), i.e. resistance to: i) initial infection, ii) spread of fungal infection, iii) kernel infection, iv) toxin accumulation, and v) resistance to yield loss/tolerance (Mesterházy, 1995). However, such separation into different types of resistance is conditional, it is more appropriate to consider them as interrelated resistance components rather than independent variables. In response to infection, different types of pathways, such as metabolic, structural, signaling, and regulatory, are activated (Mesterhazy, 2024). These pathways are highly interconnected and strongly depend on the interaction between genotype and environment (Jin et al., 2013). The penetration and spread of Fusarium fungus are more strongly influenced by environmental conditions than in biotrophic wheat diseases, such as rusts. The severity of FHB is particularly affected by weather conditions after inoculation and during the anthesis period (Miedaner et al., 2001).

Weather conditions during the flowering period strongly affect the development of Fusarium fungi (M. Buerstmayr et al., 2020). Notably, extended periods of moisture at the time of anthesis, when the temperature is close to 25 °C, can increase the risk of infection (Reis et al., 2023). Additionally, other researchers have observed a negative correlation between days to heading and FHB resistance, which can be explained by the escape of favorable weather conditions for disease development (Buerstmayr et al., 2009; Emrich et al., 2008; Parry et al., 1995). In addition to this, FHB resistance in wheat is a complex quantitative trait that is controlled by multiple minor genes and influenced by genotype-by-environment (G × E) interactions involving genetic factors of the host resistance and pathogen aggressiveness, as well as environmental conditions (Bai and Shaner, 2004; Buerstmayr et al., 2009; Kumar et al., 2019). Therefore, multi-environmental data are required to obtain reliable genetic differentiation (Mesterhazy, 2024). Consequently, wheat resistance is a result of up and down regulation of thousands of gene expressions (Buerstmayr et al., 2021). Transcriptomic studies have also revealed that genotypes with similar phenotypic resistance showed partial overlap in differentially expressed genes (DEGs) but distinct expression patterns, indicating that the defense mechanisms against FHB are multifaceted (Pan et al., 2018).

QTL mapping of FHB resistance further demonstrates genetic complexity of FHB resistance. To date, approximately 600 QTLs associated with FHB resistance have been identified in wheat (Zheng et al., 2021). Genome-wide association studies (GWAS) that enable broader allele discovery of wheat resistance to FHB than conventional QTL mapping have revealed that resistance is conferred by multiple small-to-moderate-effect marker-trait associations (MTAs), typically explaining less than 20% of the phenotypic variance (Ghimire et al., 2022; Hu et al., 2020; Wang et al., 2023; Zheng et al., 2021). Using medium- and major-effect QTLs, marker-assisted selection (MAS) can be applied to accumulate resistant alleles in one genotype (Arruda et al., 2016b). However, breeding for FHB improvement via MAS is not fully effective because of genotype-by-environment interactions and focusing on major alleles, while a considerable part of the genetic variation is conferred by small-effect markers (Yannam et al., 2025). Therefore, breeding strategies should rely on genomic selection, which simultaneously estimates all marker effects, rather than focusing only on individual QTLs to predict genomic estimated breeding values (GEBVs) (Morales et al., 2024; Verges et al., 2021). If the trait is complex and controlled by multiple loci with minor effects and epistasis is observed, genomic selection (GS) is superior to MAS. The application of specific genomic selection (GS) frameworks, such as kernel-based reproducing kernel Hilbert space (RKHS) regression models, machine learning models, and artificial neural networks, allows both additive genetic effects and nonlinear relationships among loci to be modeled, enabling the capture of epistatic interactions (Crossa et al., 2025; Jeon et al., 2026; Montesinos-López et al., 2024).

Furthermore, morphological and phenological traits associated with FHB resistance have been incorporated into genomic prediction using various approaches to improve the accuracy of FHB resistance prediction. The residual method is aimed at removing the portion of phenotypic variation in FHB that is explained by these traits. After that, genomic prediction is performed on the remaining phenotypic variation. In addition, traits associated with passive resistance can be included as additional predictors in multivariate frameworks to model all traits as predictors of FHB resistance simultaneously (Moreno-Amores et al., 2020). However, the greatest improvement in genomic prediction was observed when highly correlated traits with FHB resistance (such as heading/flowering dates or plant height) were included as covariates with fixed effects in trait-assisted genomic prediction (BLUP + Fixed Effect). Morales et al. (2024) demonstrated that including anthesis dates and plant height, together with SNP linked to the *Rht-D1* semi-dwarfing locus as covariates with fixed effects, considerably increased genomic prediction accuracy from 0.71 to 0.90 on average within three populations (Morales et al., 2024). Moreno-Amores et al. (2020) demonstrated that including heading dates as a covariate with a fixed effect, the prediction was increased from 0.75 to 0.80, while including two covariates, heading date and plant height, the prediction increased from 0.75 to 0.76 (Moreno-Amores et al., 2020).

Passive resistance is a well-studied topic, and the contribution of secondary traits to wheat resistance to FHB has been studied in numerous studies. Multiple studies have demonstrated that traits associated with passive resistance contribute to FHB resistance and can be leveraged to improve genomic prediction of wheat resistance to FHB (Berraies et al., 2023; Mao et al., 2010; Morales et al., 2024; Skinnes et al., 2008; Srinivasachary et al., 2008, Srinivasachary et al., 2009; Y. Xu et al., 2023). In our previous study, we determined that the phenotypic variation of eight morpho-phenological traits (plant height, days to heading, days to flowering, spike length, spikelets per spike, spike density, anther extrusion, and awn length) enabled the prediction of FHB resistance with an accuracy of 57-65% in cross-validation sets using random forest regression analysis. Overall, the phenotypic responses of these eight morpho-phenological traits explained from 8 to 42% of the phenotypic variance in FHB severity (Syed et al., 2026). Although passive resistance may enhance overall resistance, the relationship between SNP markers associated with active and passive resistance traits, their individual and joint contributions to the phenotypic variation of FHB resistance, and their effects on the genomic prediction accuracy remain underexplored. Previous studies have analyzed either resistance QTLs or individual morphological covariates in genomic prediction; however, to the best of our knowledge, a comparison of marker sets derived from FHB severity and passive resistance traits within the same population has not been studied. Therefore, this study evaluates these marker sets and the effect of including days to heading and anther extrusion as fixed covariates in genomic prediction models.

To address this research gap, the present study aimed to: (i) identify MTAs and QTLs significantly associated with FHB resistance and morpho-phenological traits; (ii) conduct genomic prediction using different sets of SNP markers associated with FHB severity (set 1) and morpho-phenological traits (set 2) in three genomic prediction models, KRHS, rrBLUP, and RF; (iii) determine the individual size effects and contribution of set 2 on prediction performance; and (iv) examine the effects of including phenotypic responses of days to heading (DH) and anther extrusion (AE) as covariates with fixed effects in genomic prediction. For this purpose, a genotyped wheat collection of 335 genotypes was evaluated in 2022, 2023, and 2025 for eight morpho-phenological and FHB severity traits under field conditions.

## 2 Materials and methods

### 2.1 Field trials and genotypes used

To evaluate FHB resistance, 335 genotypes of European and exotic origins were used. The set of genotypes consisted of 300 breeding lines and varieties from Nordic-Baltic countries (Norway, Estonia, Latvia, and Lithuania), which had been previously genotyped for the Nordic-Baltic wheat project, with the addition of 35 Lithuanian breeding lines (Aleliūnas et al., 2024; Lin et al., 2024). Following genetic control, three genotypes were excluded from the set. The final wheat panel used in this study consisted of 332 genotypes: 65 from Nordic countries, 181 of Baltic origin, 76 from Central and Western Europe, and 10 of exotic origin. These exotic genotypes also have well-known Asian cultivars that are resistant to FHB, that is, Sumai 3 and Wangshuibai, and the South African cultivar Gamenya was used as the susceptible check. Other well-documented resistant genotypes from CIMMYT (i.e., SHA3/CBRD and MILAN/SHA7) were also included as reference genotypes.

An alpha design with two replicates in a 1.5 m^2^ plot was used to conduct field experiments at the Institute of Agriculture, LAMMC in 2022 and 2023. Moreover, experiments were also conducted under field conditions in 2025 to record anther extrusion. For evaluation of FHB, spray inoculation was used to assess type I and overall resistance. To maintain humidity under field conditions, the bunches of spikes were covered with plastic bags for 48 hours. The details of the plant material used, inoculation techniques and concentration used, meteorological conditions during the experimental period, and evaluation of type I, II, and overall resistance were published in previous articles (Syed et al., 2026, Syed et al., 2025, Syed et al., 2024).

### 2.2 Evaluation of FHB traits

For phenotypic evaluation, the inoculated spikes were evaluated for type I, II, and overall resistance. Type I resistance or incidence was evaluated one week after inoculation. Spikes were visually assessed on the basis of FHB symptoms (**Table 1**).

**Table 1:** Evaluation criteria and timing for evaluation incidence and severity.

FHB trait	Scoring	Timing
Incidence	Presence/absence of the symptoms	1 week after inoculation
Severity	Spread of the infection within spike	2 weeks after inoculation

Each spike was carefully inspected for signs of FHB infection and counted as an infected spike, whereas spikes without any visual symptoms were counted as healthy spikes. The incidence was calculated using Equation 1:

(1) $$Incidence=\frac{Diseased\;spikes}{Healthy\;spikes}\times 100$$

To evaluate disease severity (type II), spikes were visually assessed 14 days after inoculation using a visual scale from zero “0” to “100” where 0 indicated no infection and 100 represented complete infection. The Equation 2 used to calculate the severity of infection is as follows:

(2) $$Severity=\frac{Number\;of\;infected\;spikelets}{Total\;number\;of\;spikelets\;per\;spike}\times 100$$

To determine the FHB index (overall resistance), incidence and severity data were used using the following Equation 3:

(3) $$FHBindex=\frac{Incidence\times Severity}{100}$$

The FHB index reflects the overall wheat resistance integrating both types of resistance.

### 2.3 Evaluation of phenotypic traits

Phenotypic evaluation of 335 genotypes was conducted over three growing seasons following previously described protocols (Syed et al., 2026). Phenological traits, including days to heading and flowering, were recorded as the number of days from sowing until 75% of the plants in a plot reached the heading or flowering stage.

Architectural traits comprised plant height and awn length. Plant height (cm) was measured from the soil surface to the tip of the spike, excluding awns. Awn length or presence was assessed using a visual scale (0–9), where scores reflected the degree of awn development, the higher values indicate more developed awns considering the length, shape and density.

Reproductive traits included spike length, number of spikelets per spike, spike density, and anther extrusion. Spike length (cm) was measured from the base to the tip of the spike, excluding awns. Spike density was calculated as a derived trait using the following Equation 4:

(4) $$Spike\;density=\;\frac{The\;number\;of\;spikelets}{Spike\;length}$$

Anther extrusion was visually assessed three days after flowering using a 0–5 scale, where scores represented the proportion of exerted anthers per spike (Skinnes et al., 2010).

### 2.4 Genotypic evaluation

For genotyping, a 25 K SNP chip array was used to genotype the set of genotypes as part of the NOBAL-wheat project (Aleliūnas et al., 2024; Lin et al., 2024). Genotypic data was filtered to remove monomorphic markers and individuals with abnormal heterozygosity rates. In addition, markers with more than 20% missing genotype data and with allele frequency (MAF) below 0.05 were excluded to reduce the influence of poorly genotyped markers, remove rare alleles, and improve statistical power. The filtered genotype dataset (332 genotypes and 18,417 high-quality SNP markers) was subsequently used for GWAS and genomic prediction analyses. GWAS analysis was performed, using a Bonferroni-adjusted significance threshold of P = 2.72×10^-6^, which is equal to -log_10_(P) = 5.565 (Syed et al., 2025). To account for population structure, the first two principal components (PC1 and PC2) were included as covariates in GWAS.

### 2.5 Principal component analysis (PCA)

Principal component analysis (PCA) was performed for population structure analysis using genome-wide allele variation. The first principal component explained 9.2% of the total variation, while the first two and three principal components explained 15.1% and 20.2% of the variation, respectively. According to the Elbow method the first two principal components were included as covariates. Genomic inflation factor (λ ≈ 1) additionally supported the choice of two covariates for adequate control of population structure. The details of the principal component analysis are provided in our previous article Syed et al. (2025).

### 2.6 Statistical analysis

The analysis of data was performed using R software (R-4.2.2). The Best Linear Unbiased Estimates (BLUEs) values for morpho-phenological and FHB traits across years were calculated using META-R version 6.04 (Alvarado et al., 2020). Broad-sense heritability H^2^ was calculated using “lme4” package in R. Genotypes, years, replications, and their interactions were treated as random effects in the linear mixed-effects (lme) model to calculate heritability. Broad-sense heritability (H^2^) was calculated according to the formula including multiple environments:


$${\text{H}}^{2}=\frac{Var\;(g)}{Var\;(g)\;+\;\frac{Var\;(gy)}{y}+\;\frac{Var\;(residual)}{ry}}$$

Where “g” represents genotype variance among genotypes, “gy” is the interaction between genotype x year interaction variance, y is the number of years, “r” is the number of replicates, and “ry” is the total number of observations per genotype and across years (Holland, 2003).

Genome-wide association analysis (GWAS) was performed using a Bayesian-information and Linkage-disequilibrium Iteratively Nested Keyway (BLINK) model with the first two principal components as covariates in the R package “GAPIT3” (Wang and Zhang, 2021).

Marker-trait associations were considered significant at Bonferroni-adjusted threshold (α=0.05/18,417, that is equal to P = 2.71×10^-6^). This threshold was further converted into a-log10 (*P*) value of 5.565.

The pairwise linkage disequilibrium (LD) among SNP markers was determined for each chromosome using the full-matrix option in “TASSEL 5” (Bradbury et al., 2007). The nonlinear model of Marroni et al. (2011) was applied to summarize the relationship between LD decay and physical distance (Marroni et al., 2011). Subsequently, a half-decay distance based on the maximum LD R^2^ value was determined.

Spearman’s rank correlation analysis was performed to analyse the correlation between the number of resistant alleles and genotypes’ resistance to FHB.

Genomic prediction was performed using set 1 (FHB-associated SNPs) and set 2 (morpho-phenological trait-associated SNPs) in three models: Reproducing Kernel Hilbert Space (RKHS), Ridge Regression Best Linear Unbiased Prediction (rrBLUP), and Random Forest regression (RF). For most analyses, set 1, which comprised 48 FHB-associated SNPs identified by conventional GWAS in GAPIT3 (11 MTAs from this study and 37 from Syed et al., 2025), was used. Set 2 comprised SNPs significantly associated with morpho-phenological traits from conventional GWAS in GAPIT3. To reduce preselection bias, set 1 was redefined using cross-validated BLINK GWAS in GAPIT4 (5-fold CV × 40 repeats). This CV-derived set 1 was used for trait-assisted genomic prediction. For trait-assisted genomic prediction, days to heading and anther extrusion were included as fixed-effect covariates in rrBLUP and RKHS models (Endelman, 2011; Morales et al., 2024). Using this approach, it became possible to evaluate whether passive-associated traits improve prediction accuracy compared to predictions based solely on the marker sets. rrBLUP was performed using the rrBLUP package (Endelman, 2011). In RKHS, three Gaussian kernels with bandwidths h/5, h, and 5h were fitted in BGLR with 10,000 MCMC iterations, a burn-in of 2,000, and thinning interval of 5, with DH and AE included as fixed effects in covariate models Pérez and de los Campos, 2014. In RF, DH and AE were included as additional predictors together with SNP markers, using Ranger package via mlr3 with 2,000 trees and hyperparameters (mtry, min.node.size, sample.fraction, replace, and splitrule) optimized by random search (100 replicates) with 5-fold cross-validation on the basis of search for minimal RMSE (Wright and Ziegler, 2017). Accuracy of prediction was evaluated using 100 replications of Monte Carlo cross-validation (80% training, 20% validation) with set.seed(123) for reproducibility, in each replication. To evaluate predictive performance, Pearson’s correlation (*r*) between observed and predicted values was calculated. The coefficient of determination was calculated as the square of Pearson’s correlation coefficient (r^2^), representing the proportion of phenotypic variance explained (Shan, 2022).

To reduce overfitting associated with marker preselection in the complete dataset of genotypes, set 1 was additionally defined using cross-validated GWAS for genomic predictions. For this purpose, a cross-validated BLINK GWAS was performed in GAPIT4. 5-fold cross-validation was used to randomly partition genotypes 40 times. In each fold, GWAS was conducted on the training set only (Wang and Zhang, 2026).

## 3 Results

Overall, 335 genotypes were evaluated for eight different morphological traits and FHB severity in 2022, 2023, and 2025. Trait responses were evaluated against FHB under field conditions using the spray-inoculation technique. For heritability estimates, replicates from each year were used for morphological and phenological traits. The average heritability for these traits was 0.75, ranging from medium to extremely high heritability (0.50 to 0.94). The highest heritability was shown by the trait awn length, that is 0.94, whereas the lowest heritability among the traits was recorded for anther extrusion (0.50) (**Table 2**).

**Table 2:** Heritability estimates for morphological traits across multiple years.

Traits	H^2^
Anther extrusion	0.50
Awn length	0.94
Days to flowering	0.69
Days to heading	0.84
Plant height	0.81
Spike length	0.75
Spikelets/spike	0.82
Spike density	0.78
FHB Severity	0.66

### 3.1 GWAS analysis for morpho-phenological and FHB resistant MTAs

GWAS was conducted to identify marker-trait associations (MTAs) for the individual morpho-phenological traits and FHB resistance and their contribution under different environments. In total, 118 MTAs with known positions and 17 MTAs with unknown positions were detected. These MTAs were identified on all 21 chromosomes and were distributed on all three sub-genomes (A, B, and D genomes). The A genome included 45 MTAs, 56 were found on the B genome, and 17 were found on the D genome. A significant portion of the variation was explained by the A and B genomes, which had the greatest number of MTAs.

Moreover, a total of 11 MTAs with eight known and three unknown positions were detected for FHB resistance, which were located on sub-genomes A and B. However, no SNP associated with disease severity were found in the D genome.

### 3.2 Major *QTLs* associated with morphological traits

Based on the half-decay distance of individual chromosomes, the physical intervals of morpho-phenological and FHB QTLs were defined (**Supplementary Table 1**). Overall, 28 major QTLs were defined on the basis of a higher coefficient of determination (R^2^ >10%) (**Supplementary Tables 1**, **2**). Among them, most belonged to morpho-phenological traits (24 QTLs), and four QTLs belonged to FHB resistance.

The strongest QTL “*QAL-5A*” (BobWhite_c8266_227) was identified on chromosome 5A with a significant effect in three trials on awn length, showing the highest coefficient of determination of approximately 70 to 90%. The SNP markers for other major morpho-phenological traits showed a range of R^2^ values from 6.3 to 16.5%, and listed in **Supplementary Table 1**.

To evaluate the physical overlap of our identified QTLs with previously reported FHB-associated QTLs, we used consolidated QTLs from 113 articles that were aligned with the Chinese Spring reference genome RefSeq V1.1 by Zheng et al. (2021). Among the 24 major QTLs, 11 QTLs associated with morpho-phenological traits overlapped with reported FHB-resistant QTLs.

The QTL for anther extrusion (*QAE_4A*) was identified on chromosomes 4A and was found in previously reported physical intervals from 632.86 to 684.62 Mb (Petersen et al., 2017a; Zhang et al., 2018; Zheng et al., 2021). The QTL for awn length (*QAL_5A*), presented on chromosome 5A, also falls in the previously reported physical positions, that is, 688.14–698.19 Mb reported by several researchers (Holzapfel et al., 2008; Malihipour et al., 2017; Paillard et al., 2004; Zheng et al., 2021). In addition, the physical positions identified in this study for the trait days to heading on 6B and 7A were colocalized with previously reported physical intervals (430.08–641.01 Mb) associated with FHB resistance (Kumar et al., 2007; Li et al., 2011; Sari et al., 2018; Yu et al., 2008; Zheng et al., 2021). The physical positions identified for plant height in this study were located on chromosomes 3A and 4A and overlapped with previously reported FHB-resistant physical intervals, that is, 475.73–484.10 Mb and 708.60–743.92 Mb on 3A and 4A, respectively (Buerstmayr and Buerstmayr, 2015; Mardi et al., 2006; Petersen et al., 2017b; Ren et al., 2016; Szabó-Hevér et al., 2012; Zheng et al., 2021).

QTLs for spike density (*QSD_3A)*, spike length (*QSL_1B*), and spikelets per spike (*QSS_4A* and *QSS_5B*) also colocalized with previously reported intervals (**Supplementary Table 1**) (Zheng et al., 2021).

The quantitative trait locus *QDH/DF_7B* was associated with two traits: days to heading (DH) and days to flowering (DF). *QDF/SD_7D* was associated with days to flowering (DF) and spike density (SD), and *DF/PH_3B* with days to flowering (DF) and plant height (PH). Shared QTLs among heading, flowering, plant height and spike traits suggest a pleiotropic effect or close linkage of their loci (**Supplementary Table 1**).

### 3.3 Major *QTLs* associated with FHB severity

Further, 11 QTLs were identified for severity 2025 and combined severity of three years (2022, 2023, and 2025), as presented in **Supplementary Table 2**. Two highly significant QTLs on chromosomes A and B showed more than a 10% coefficient of determination (R^2^). However, no major QTLs were observed on chromosome D. Additionally, two QTLs were defined with R^2^ <1%.

Although the set of SNP markers associated with FHB severity was unique compared to the morpho-phenological SNP markers, one marker was in linkage disequilibrium. The SNP marker Excalibur_c29707_318 (411.87–413.00 Mb) was located close to the SNP marker RAC875_c400_1271 (411.87–413.45 Mb), which is associated with anther extrusion.

Moreover, five significant QTLs identified in this study for FHB resistance were found to be colocalized with previously reported QTLs. The QTLs (*QFHB_1B, QFHB_2B, QFHB_3B* and *QFHB_7B*) were identified on chromosomes 1B (446.45–448.00 Mb), 2B (646.32–648.00 Mb), 3B (647.71–650.00 Mb and 824.42–826.00 Mb), and 7B (712.32–713.00 Mb) for FHB resistance were overlapped with physical intervals identified by several researchers previously (Bourdoncle and Ohm, 2003; Draeger et al., 2007; Eckard et al., 2015; Giancaspro et al., 2016; Jia et al., 2005; Lin et al., 2004; Lu et al., 2013b; Szabó-Hevér et al., 2012; Yi et al., 2018; Zhang et al., 2018; Zhou et al., 2004).

Overall, eight QTLs associated with FHB resistance were not new because they overlapped with previously reported physical intervals. However, three FHB-related QTLs were not co-located in any previously defined physical interval and are presumed to be newly identified regions. However, further validation is required.

### 3.4 Correlation between the number of resistance alleles associated with FHB resistance and morpho-phenological traits and genotype resistance

Individual SNPs associated with morpho-phenological traits were not detected as significantly associated with FHB resistance using GWAS, whereas these SNPs might still contribute to FHB resistance. To measure the overall relationships between SNP markers associated with FHB resistance and morpho-phenological traits, Spearman’s correlation analysis between the number of resistant alleles identified in the GWAS and genotype resistance was performed. Eleven MTAs significantly associated with FHB severity in this study and 37 from our previous study were jointly included in set 1. In total, 87 MTAs significantly associated with morpho-phenological traits were identified in the GWAS and included in the analysis as set 2.

**Figure 1:**
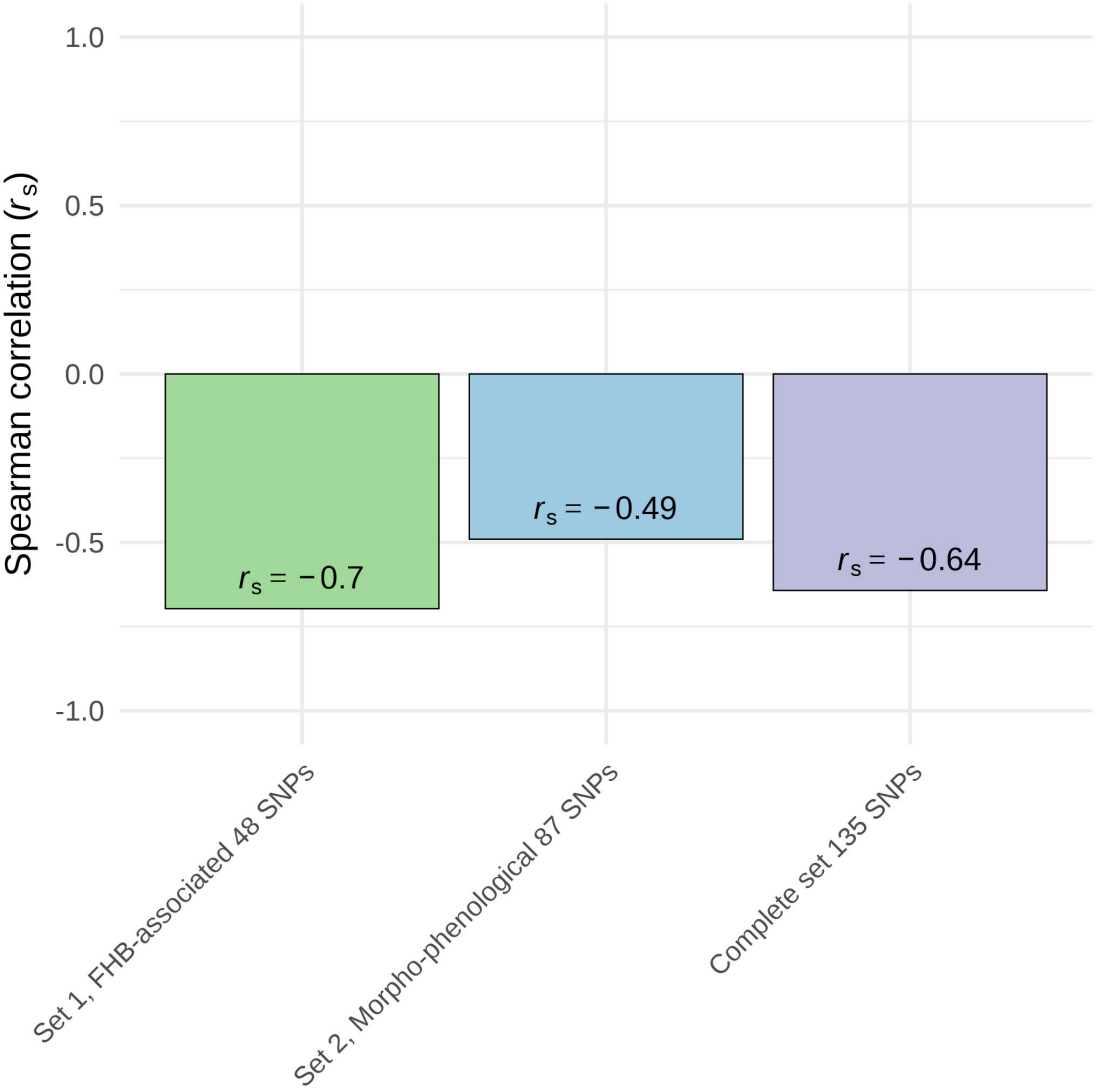
Correlation analysis between the number of resistant alleles and genotypes’ resistance to FHB across different marker sets.

The number of resistant alleles in set 1 and genotype FHB severity (BLUEs over three years) were significantly correlated (*r_s_* = −0.7, *p* < 0.001). The correlation between the number of resistant alleles associated with morphological traits (set 2) and FHB severity was moderate (*r_s_* = −0.49, *p* < 0.001). Furthermore, combining the markers of set I with those of set II slightly reduced the correlation (*r_s_* = -0.64, *p* < 0.001) compared to the correlation with set I alone (**Figure 1**). This suggests that information from SNP markers associated with morpho-phenological traits is more redundant than meaningful for prediction. Morpho-phenological traits may influence FHB indirectly through avoidance of favourable conditions (for example: higher distance from soil or coincidence between flowering and weather conditions). Therefore, redundancy of confounded genetic information could be introduced after merging two SNP sets.

### 3.5 Genomic prediction for FHB severity

#### 3.5.1 Proportion of variance explained by separate marker sets in genomic prediction models for FHB severity

**Figure 2:**
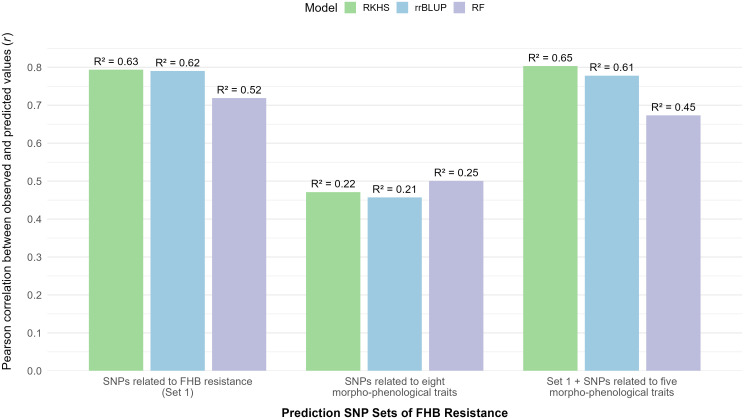
Comparison of the predictive performances of different SNP sets across the three models.

Several prediction models were applied to determine the individual and combined contributions of the two marker sets to FHB severity: Reproducing Kernel Hilbert Space (RKHS) regression, Ridge Regression Best Linear Unbiased Prediction (rrBLUP), and Random Forest Regression (RF). In all models, the performance of individual marker sets and their combinations was evaluated using out-of-sample predictions based on 100 repeated cross-validation runs. Model accuracy was assessed using the Pearson correlation coefficient (*r*) and coefficient of determination (R^2^), which demonstrates the proportion of variance explained by the model. Using set 1, the prediction accuracy was the highest in RKHS (r = 0.79; R^2^ = 0.63) and close in rrBLUP (r = 0.76; R^2^ = 0.62) and slightly less in RF (r = 0.72; R^2^ = 0.52). Set 2 demonstrated lower predictive accuracy than set 1 (r = 0.47, R2 = 0.22 for RKHS; r = 0.46, R^2^ = 0.21 for rrBLUP; r = 0.50, R^2^ = 0.25 for RF). The coefficient of determination (R^2^) indicated that set 1 explained 63%, 62%, and 52% of the phenotypic variation (FHB severity) in the RKHS, rrBLUP, and RF models, respectively. The second set of 87 SNP markers, which were significantly associated with morpho-phenological traits, alone explained 22%, 21%, and 25% of FHB severity in the RKHS, rrBLUP, and RF models, respectively. The RKHS and rrBLUP models outperformed the RF model in predictive accuracy when set 1 was included alone and when the two sets were combined. The accuracy was improved and the model explained greater phenotypic variation (2% of absolute increase), when two marker sets were combined in the RKHS model, while performance declined slightly in the rrBLUP and RF models, with absolute decreases of 1% and 7%, respectively (**Figure 2**).

**Figure 3:**
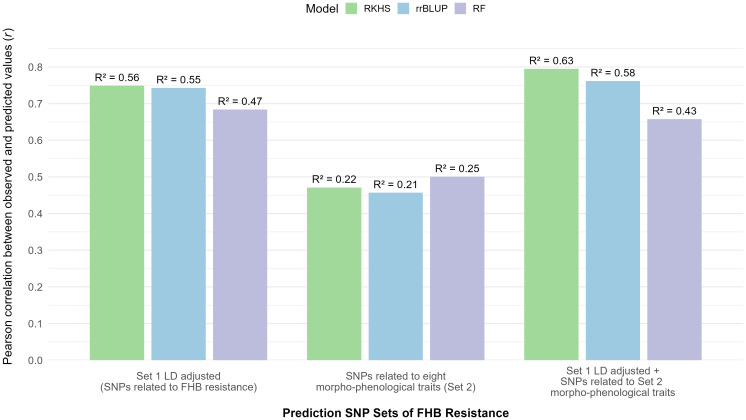
Comparison of predictive performances of different SNP sets across three models after LD adjustment.

Although no overlapping markers were found between the two sets, 13 SNP markers from set 1 were in Linkage Disequilibrium (LD) with SNPs from set 2. A stronger complementary effect of SNPs from set 2 was observed when markers that were in LD linkage were removed from set 1. After excluding LD-linked markers from set 1, the contribution of set 2 was larger, increasing by 7% in the RKHS and 3% in the rrBLUP models. However, merging of set 1 with set 2 did not result in any increase in the RF model, while the RF model demonstrated advantageous performance for individual set 2 (**Figure 3**).

#### 3.5.2 Genomic prediction for FHB severity with phenotypic responses as covariates

**Figure 4:**
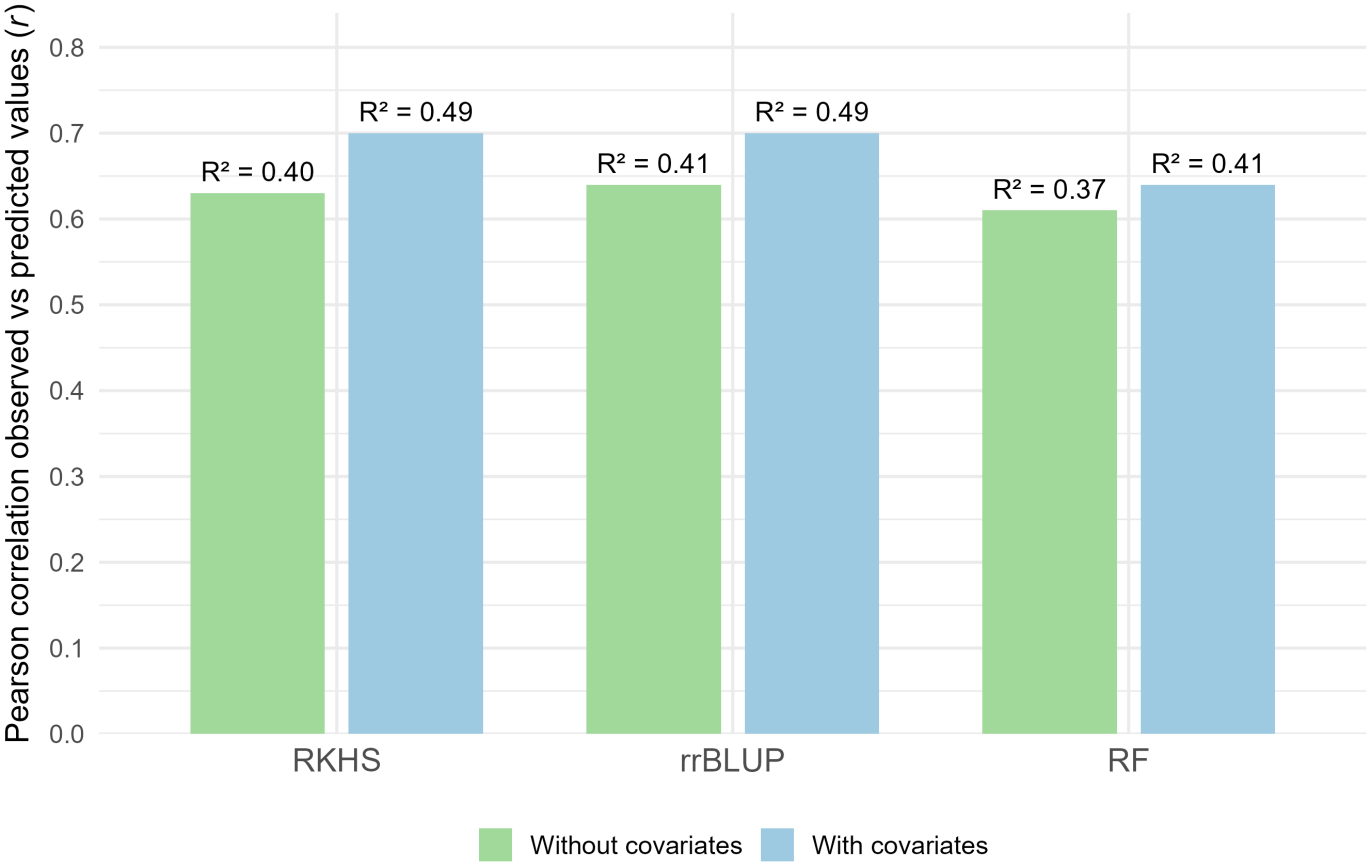
Comparison of the models after including days to heading (DH) and anther extrusion (AE) as covariates using SNPs identified by GWAS on the training set (80% of lines) in 200 cross-validation runs (5 fold × 40 replicates).

Additionally, genomic prediction was performed, including the phenotypic responses of two traits, days to heading and anther extrusion, as covariates with a fixed effect into CV-derived set 1 in the RKHS and rrBLUP models. In the RF model, these traits were included as additional predictors. After including these phenotypic responses, the genomic prediction accuracy (*r*) improved by 7% in RKHS, by 6% in rrBLUP and by 3% in RF. The ranking of the models was not affected (**Figure 4**).

#### 3.5.3 Genomic prediction for FHB severity, based on the whole set of markers

**Figure 5:**
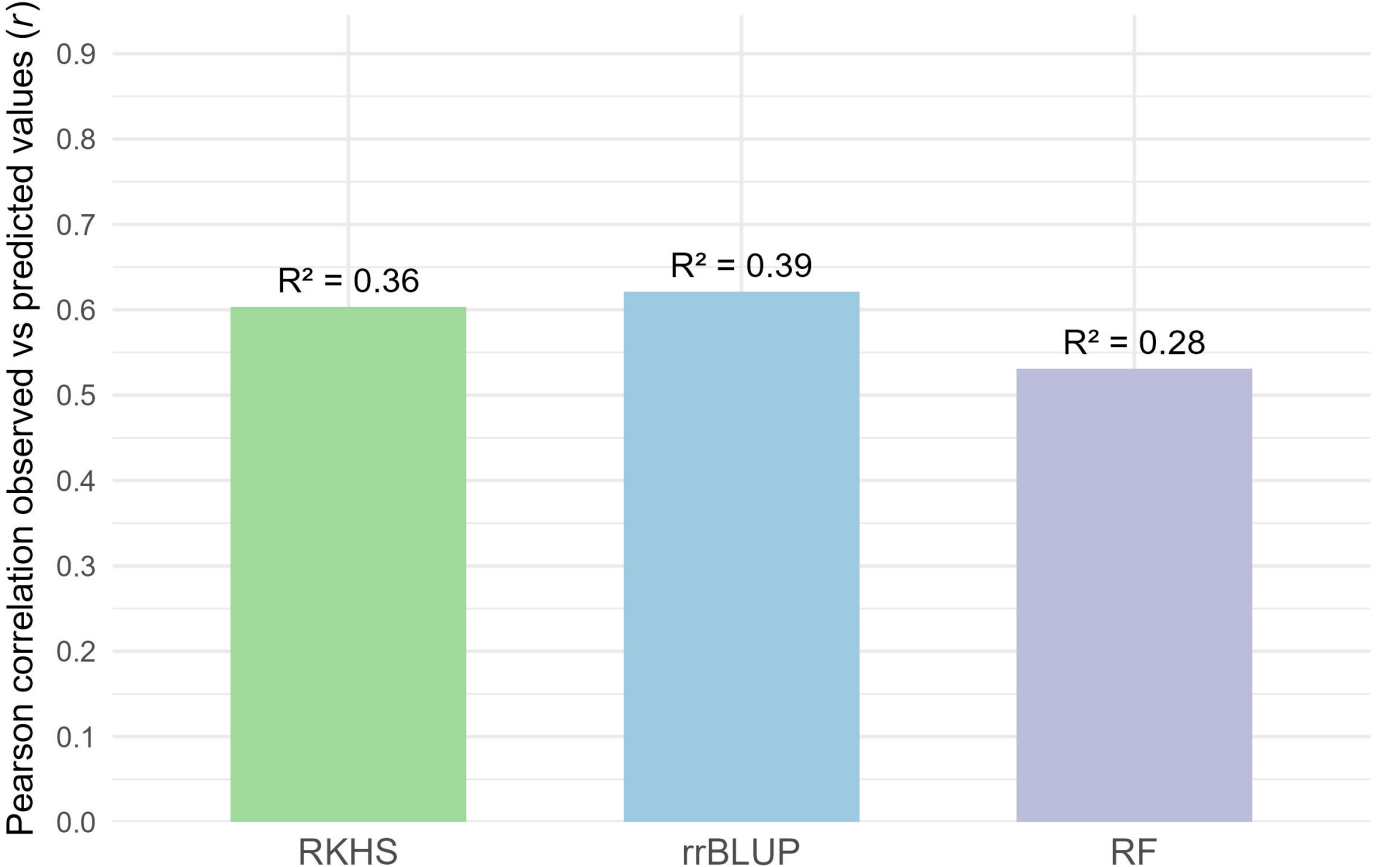
Genomic prediction on the basis of a complete set of markers (18,417 SNPs) across three models.

Finally, genomic prediction was performed using an entire set of markers retained after quality filtering (18,417 SNPs). Including all markers resulted in a decrease in prediction accuracy. The prediction accuracy, measured by Pearson’s correlation coefficient (r), was 0.60 for RKHS, 0.62 for rrBLUP, and 0.53 for RF, the corresponding R^2^ values were 0.36, 0.39 and 0.28, respectively (**Figure 5**).

## 4 Discussion

Passive resistance is an essential component of overall wheat resistance, constituting the first layer of defense and enhancing active resistance (Gao et al., 2024; Gorash et al., 2021). In our study, we examined the associations between eight morpho-phenological traits i.e., plant height, days to heading, flowering, spike length, spikelet number, spike density, awn length, and anther extrusion, and FHB severity. Because passive resistance is associated with morpho-phenological traits and is considered the first layer of resistance, these associations provide breeders with additional valuable criteria for effective selection, especially during the initial breeding stages (Steiner et al., 2017).

### 4.1 Heritability estimates of morphological and FHB traits

Broad-sense heritability analysis of morpho-phenological traits demonstrated a different range of H^2^ values from 0.50 to 0.94. In our study, the highest heritability was observed for awn length. Long awns may potentially retain FHB spores, which can subsequently be redistributed to nearby florets by dew droplets (Mesterházy, 1995). Although their role in providing resistance against FHB is contradictory. Giancaspro et al. (2016) reported that awned genotypes are more resistant (Giancaspro et al., 2016), whereas other studies have shown that genotypes with awns are more susceptible (Mesterházy, 1995). Our findings are consistent with previous reports, other researchers have also reported high heritability scores for awn length (Feyisa et al., 2025; Würschum et al., 2020). The QTL identified in our study for awn length was located on chromosome 5A (698.508 Mb), which is located 0.02 Mb away from the candidate gene *TraesCS5A02G542800* of the *B1* and consistent with previously FHB-related major QTL (*Qfhb.spa-5A.1*) of FHB resistance on chromosome 5A (Bazhenov et al., 2025; DeWitt et al., 2020; Berraies et al., 2023). Similarly, other morphological traits also showed significantly high heritability estimates, such as days to heading, plant height, and number of spikelets (i.e., 0.84, 0.81, and 0.82, respectively), which is consistent with the results of Thakur et al. (2025). Furthermore, our results exhibited moderate-to-high heritability for FHB resistance (0.66). The heritability estimates were consistent with other research findings, which also observed moderate-to-high heritability estimates (Buerstmayr et al., 2000; Nannuru et al., 2022). Highly heritable morpho-phenological traits can be applied more easily in breeding, while moderate heritability traits such as anther extrusion should be evaluated across multiple environments for reliable breeding.

### 4.2 Identification of MTAs and QTLs associated with FHB resistance and morpho-phenological traits

GWAS of the genetic architecture of wheat resistance to FHB demonstrated that resistance is quantitative and conferred by many small-effect loci. Ghimire et al. (2022) identified 160 MTAs associated with FHB and 11 with plant height. Among them, 11 QTLs showed major effects and explained >10% of the phenotypic variation in FHB resistance (Ghimire et al., 2022). Wang et al. (2023) identified 66 MTAs associated with FHB resistance and morphological traits, which explained phenotypic variations ranging from 5.4 to 11.2%. FHB resistance is controlled by numerous small-effect loci, which is consistent with the findings of our study.

On the basis of GWAS findings, two sets of markers were selected. Set 1 consisted of SNP markers significantly associated with FHB resistance, and set 2 consisted of SNP markers associated with eight morpho-phenological traits. The first set of markers already included all significant signals related to FHB resistance and may contain markers associated with both active and passive resistance. The markers in the second set did not demonstrate a significant association with FHB individually. Nonetheless, individual SNPs associated with morpho-phenological traits were not detected as significantly related to FHB resistance in GWAS, but these SNPs may still be related to FHB resistance. Although a direct overlap between markers was not found, 13 markers from set 1 could be linked to morpho-phenological markers by linkage disequilibrium according to their physical positions. Therefore, the size effect of set 1 may have been enhanced by passive resistance alleles through the LD linkage. Excluding LD-linked markers from set 1, the contribution of markers from set 2 was 7 and 3% after combining the two sets in the RKHS and rrBLUP models, respectively, while without LD adjustment, set 2 improved the explanation of phenotypic variation only in RKHS by 2%.

We identified 87 MTAs associated with eight morpho-phenological traits. Among them, 24 demonstrated major effects (R^2^ above 10%). However, it should be noted that some MTAs may also be false positives or represent only environment-specific associations and therefore require validation in different populations and environments. Comparing the physical regions of major morphological QTLs, we found that 11 of 24 colocalized with previously identified FHB-related QTLs. The colocalization of SNPs associated with morpho-phenological traits and previously identified FHB-resistance QTLs indicates close physical proximity and possible LD linkage but does not confirm their causality. However, this overlap supports the importance of morpho-phenological traits in breeding for FHB resistance. Our previous study demonstrated that the phenotypic responses of the eight key traits explained up to 42% of the variance in FHB severity. The accuracy of the prediction ranged from 57 to 65%, depending on the year conditions, using random forest regression analysis under a cross-validation scheme (Syed et al., 2026). Therefore, it may be assumed that the alleles from set 2 may have small individual effects that are masked by the environmental effects. Moreover, although these markers may have weak individual effects, they can collectively contribute through additive interactions. In addition, GWAS algorithms could not detect them as significantly associated because these markers could be involved in epistatic or other nonlinear interactions.

### 4.3 Genomic prediction for wheat resistance to FHB

Genomic selection (GS) is an effective approach to assist breeding for complex traits with small-effect loci (Jonas et al., 2019). The genetic architecture of wheat resistance indicates that genomic selection, which may consider the additive effects of small-effect markers, is more advantageous than marker-assisted selection (MAS), which considers only major effect markers. The research studies of Arruda et al. (2016a) suggested that the prediction accuracy for FHB resistance was medium-to-high for genomic selection compared with MAS, which yielded lower prediction values (Arruda et al., 2016a).

In this study, genomic prediction was performed using the entire set of markers, as well as separate marker sets 1 and 2, which consisted of markers associated with FHB resistance and eight morpho-phenological traits identified through GWAS. Additionally, phenotypic responses (DH and AE) were included as fixed covariates in genomic prediction models based on marker set 1 in RKHS and rrBLUP and as additional predictors in RF model. These approaches were used to measure the contribution of morpho-phenological traits to FHB resistance and to select the best-performing scenario for the accurate prediction of FHB resistance. It is noteworthy that genomic prediction for FHB resistance was significantly improved when models were based on significantly associated GWAS markers rather than the entire set of markers. The prediction accuracy was considerably increased in all three models when SNP markers from cross-validated GWAS were used for prediction instead of 18,417. Set 1 of markers was selected by using cross-validated GWAS, with 5-fold cross-validation repeated 40 times, to avoid leakage of the data and to ensure that GWAS performed on training folds only. This approach was used to reduce preselection bias (the same population and training of full set of genotypes). Genomic prediction was then performed using RKHS, rrBLUP and RF models (**Figure 4**). However, it is important to mention that this set of SNP markers was identified using different types of resistance (incidence, severity, and FHB index) across three years of field trials (Syed et al., 2025).

To measure the joint contribution of markers from sets 1 and 2 to FHB resistance and to compare the genetic patterns in these sets, RKHS, rrBLUP and RF models were used. rrBLUP is a widely used model and one of the most effective models for detecting associations between genetic markers and phenotypic responses (Arruda et al., 2015). Random Forest regression (RF) analysis has the advantage of revealing nonlinear interactions (Goldstein et al., 2011; Salman et al., 2024). However, RF does not explicitly model the linkage disequilibrium effects between SNPs in genetic analyses. In our study, RF was less effective than rrBLUP and RKHS. Surprisingly, the prediction accuracy of the rrBLUP and RF models was not improved by combining the two sets of markers. This may indicate that set 2 possess limited amount of additional meaningful information for prediction. Because morpho-phenological traits are correlated traits and contribute indirectly to FHB resistance, this may indicate LD overlaps, redundancy or confounding information from set 2 for FHB prediction. RKHS regression is designed for the genetic analysis of complex traits with nonlinear interactions. The prediction slightly increased when the two sets were treated as separate Gaussian kernels with multiple bandwidths (h/5, h, and 5h). This allowed the model to weigh each kernel according to the set’s contribution to the phenotypic variance.

Furthermore, the phenotypical responses of passive-related traits (days to heading and anther extrusion) were included in genomic prediction models as covariates with fixed effects in the RKHS and rrBLUP models and as additional predictors in the RF model. Adding phenotypic responses as fixed effect covariates increased R^2^ by 0.09 (9 percentage points) in the RKHS, and by 0.08 (8 percentage points) in rrBLUP. In RF, including these traits as additional predictors increased R^2^ by 0.04 (4 percentage points) (**Figure 4**). The greater contribution of fixed covariates compared to significantly associated SNPs may be attributed to the type and complexity of the information. Phenotypic responses present a single-channel signal, whereas SNP markers provide dispersed signals across multiple alleles.

## 5 Conclusion

In conclusion, the accuracy of genomic prediction for wheat resistance to FHB was substantially improved leveraging cross-validated GWAS and exploiting passive resistance in this study. Genomic selection may be enhanced, incorporating significantly associated markers from cross-validated GWAS identified through multi-environment trials. SNP markers significantly associated with passive resistance can be included as a separate Gaussian kernel in the multiple-kernel RKHS model. Additionally, the phenotypic responses of traits associated with passive resistance can be included as fixed covariates in the rrBLUP or RKHS models. Secondary traits may assist in wheat breeding for FHB resistance. Although traits associated with passive resistance can significantly improve resistance, their use has practical limitations, and breeders should consider possible negative consequences. Phenotyping of anther extrusion should be done precisely at the same stage of anthesis, which is difficult to implement when flowering dates differ among genotypes, especially during early generation selection. Using this approach over multiple breeding cycles, a population will be shifted into late-ripening and greater anther extrusion using the trait-assisted selection. Although shifting toward more late-ripening genotypes should be considered, breeding for fully extruded genotypes can be extensively exploited. Nevertheless, the validation of these findings across different populations and environments is still required. In future studies, FHB severity and passive-resistance traits should be integrated in multi-trait genomic selection and evaluated across different populations and environments. In addition, the identified QTLs should be functionally validated, for example, using transcriptomic studies with candidate-gene analysis and further gene knockout approaches to confirm their role in FHB resistance.

## Data Availability

The original contributions presented in this study are included in the **Supplementary Material**. Further inquiries can be directed to the corresponding author.

## References

[B1] Aleliūnas A. Gorash A. Armonienė R. Tamm I. Ingver A. Bleidere M. . (2024). Genome-wide association study reveals 18 QTL for major agronomic traits in a Nordic–Baltic spring wheat germplasm. Front. Plant Sci. 15. doi: 10.3389/fpls.2024.1393170 38974985 PMC11224466

[B2] Alvarado G. Rodríguez F. M. Pacheco A. Burgueño J. Crossa J. Vargas M. . (2020). META-R: A software to analyze data from multi-environment plant breeding trials. Crop J. 8, 745–756. doi: 10.1016/j.cj.2020.03.010 38826717

[B3] Arruda M. P. Brown P. Brown-Guedira G. Krill A. M. Thurber C. Merrill K. R. . (2016a). Genome-wide association mapping of Fusarium head blight resistance in wheat using genotyping-by-sequencing. Plant Genome 9, 1–11. doi: 10.3835/plantgenome2015.04.0028 27898754

[B4] Arruda M. P. Brown P. J. Lipka A. E. Krill A. M. Thurber C. Kolb F. L. (2015). Genomic selection for predicting Fusarium head blight resistance in a wheat breeding program. Plant Genome 8, plantgenome2015.01.0003. doi: 10.3835/plantgenome2015.01.0003 33228272

[B5] Arruda M. P. Lipka A. E. Brown P. J. Krill A. M. Thurber C. Brown-Guedira G. . (2016b). Comparing genomic selection and marker-assisted selection for Fusarium head blight resistance in wheat (Triticum aestivum L.). Mol. Breed. 36, 84. doi: 10.1007/s11032-016-0508-5 30311153

[B6] Bai G. Shaner G. (2004). Management and resistance in wheat and barley to Fusarium head blight. Annu. Rev. Phytopathol. 42, 135–161. doi: 10.1146/annurev.phyto.42.040803.140340 15283663

[B7] Bazhenov M. Bespalova L. Samarina M. Polevikova N. Agaeva E. Debeliy A. . (2025). Genome-wide association study of agronomic traits in winter wheat (Triticum aestivum L.) using a custom SNP marker set. BMC Plant Biol. 25, 1279. doi: 10.1186/s12870-025-07322-y 41034704 PMC12487310

[B8] Berraies S. Cuthbert R. Knox R. Singh A. DePauw R. Ruan Y. . (2023). High-density genetic mapping of Fusarium head blight resistance and agronomic traits in spring wheat. Front. Plant Sci. 14. doi: 10.3389/fpls.2023.1134132 37284725 PMC10241073

[B9] Bourdoncle W. Ohm H. W. (2003). Quantitative trait loci for resistance to Fusarium head blight in recombinant inbred wheat lines from the cross Huapei 57-2 / Patterson. Euphytica 131, 131–136. doi: 10.1023/A:1023056207513 41886696

[B10] Bradbury P. J. Zhang Z. Kroon D. E. Casstevens T. M. Ramdoss Y. Buckler E. S. (2007). TASSEL: software for association mapping of complex traits in diverse samples. Bioinformatics 23, 2633–2635. doi: 10.1093/bioinformatics/btm308 17586829

[B11] Buerstmayr H. Ban T. Anderson J. A. (2009). QTL mapping and marker-assisted selection for Fusarium head blight resistance in wheat: a review. Plant Breed. 128, 1–26. doi: 10.1111/j.1439-0523.2008.01550.x 40046247

[B12] Buerstmayr H. Steiner B. Lemmens M. Ruckenbauer P. (2000). Resistance to Fusarium head blight in winter wheat: Heritability and trait associations. Crop Sci. 40, 1012–1018. doi: 10.2135/cropsci2000.4041012x

[B13] Buerstmayr M. Buerstmayr H. (2015). Comparative mapping of quantitative trait loci for Fusarium head blight resistance and anther retention in the winter wheat population Capo × Arina. Theor. Appl. Genet. 128, 1519–1530. doi: 10.1007/s00122-015-2527-8 25982129 PMC4477076

[B14] Buerstmayr M. Steiner B. Buerstmayr H. (2020). Breeding for Fusarium head blight resistance in wheat—Progress and challenges. Plant Breed. 139, 429–454. doi: 10.1111/pbr.12797 40046247

[B15] Buerstmayr M. Wagner C. Nosenko T. Omony J. Steiner B. Nussbaumer T. . (2021). Fusarium head blight resistance in European winter wheat: Insights from genome-wide transcriptome analysis. BMC Genomics 22, 470. doi: 10.1186/s12864-021-07800-1 34167474 PMC8228913

[B16] Crossa J. Martini J. W. R. Vitale P. Pérez-Rodríguez P. Costa-Neto G. Fritsche-Neto R. . (2025). Expanding genomic prediction in plant breeding: Harnessing big data, machine learning, and advanced software. Trends Plant Sci. 30, 756–774. doi: 10.1016/j.tplants.2024.12.009 39890501

[B17] DeWitt N. Guedira M. Lauer E. Sarinelli M. Tyagi P. Fu D. . (2020). Sequence-based mapping identifies a candidate transcription repressor underlying awn suppression at the B1 locus in wheat. New Phytol. 225, 326–339. doi: 10.1111/nph.16152 31465541 PMC6916393

[B18] Draeger R. Gosman N. Steed A. Chandler E. Thomsett M. Srinivasachary . (2007). Identification of QTLs for resistance to Fusarium head blight, DON accumulation and associated traits in the winter wheat variety Arina. Theor. Appl. Genet. 115, 617–625. doi: 10.1007/s00122-007-0592-3 17607557

[B19] Eckard J. T. Gonzalez-Hernandez J. L. Caffe M. Berzonsky W. Bockus W. W. Marais G. F. . (2015). Native Fusarium head blight resistance from winter wheat cultivars ‘Lyman,’ ‘Overland,’ ‘Ernie,’ and ‘Freedom’ mapped and pyramided onto ‘Wesley’-Fhb1 backgrounds. Mol. Breed. 35, 6. doi: 10.1007/s11032-015-0200-1 30311153

[B20] Emrich K. Wilde F. Miedaner T. Piepho H. P. (2008). REML approach for adjusting the Fusarium head blight rating to a phenological date in inoculated selection experiments of wheat. Theor. Appl. Genet. 117, 65–73. doi: 10.1007/s00122-008-0753-z 18392606

[B21] Endelman J. B. (2011). Ridge regression and other kernels for genomic selection with R package rrBLUP. Plant Genome 4, 250–255. doi: 10.3835/plantgenome2011.08.0024

[B22] European Commission (2023). Using Less Chemical Pesticides: European Commission Publishes Toolbox of Good Practices - Agriculture and Rural Development. Available online at: https://agriculture.ec.europa.eu/media/news/using-less-chemical-pesticides-european-commission-publishes-toolbox-good-practices-2023-02-28_en.

[B23] FAOSTAT (2026). Available online at: https://www.fao.org/faostat/en/#data/QCL/visualize (Accessed November 14, 2025).

[B24] Feyisa G. Singh B. C. S. Nepir G. (2025). Genetic Variability and Heritability and Yield Related Traits among Bread Wheat (*Triticum aestivum* L.) Accessions in West Shewa, Central Ethiopia. 40 (2), 1037–1050.

[B25] Gao X. Li F. Sun Y. Jiang J. Tian X. Li Q. . (2024). Basal defense is enhanced in a wheat cultivar resistant to Fusarium head blight. J. Integr. Agric. 23, 1238–1258. doi: 10.1016/j.jia.2023.06.014 38826717

[B26] Ghimire B. Mergoum M. Martinez-Espinoza A. D. Sapkota S. Pradhan S. Babar M. A. . (2022). Genetics of Fusarium head blight resistance in soft red winter wheat using a genome-wide association study. Plant Genome 15, e20222. doi: 10.1002/tpg2.20222 35633121 PMC12806919

[B27] Giancaspro A. Giove S. L. Zito D. Blanco A. Gadaleta A. (2016). Mapping QTLs for Fusarium head blight resistance in an interspecific wheat population. Front. Plant Sci. 7. doi: 10.3389/fpls.2016.01381 27746787 PMC5040704

[B28] Goldstein B. Polley E. Briggs F. (2011). Random forests for genetic association studies - PMC. PMC. 10 (1). doi: 10.2202/1544-6115.1691 PMC3154091 22889876

[B29] Gorash A. Armonienė R. Kazan K. (2021). Can effectoromics and loss-of-susceptibility be exploited for improving Fusarium head blight resistance in wheat? Crop J. 9, 1–16. doi: 10.1016/j.cj.2020.06.012 38826717

[B30] Graham S. Browne R. A. (2009). Anther extrusion and Fusarium head blight resistance in European wheat. J. Phytopathol. 157, 580–582. doi: 10.1111/j.1439-0434.2008.01524.x 40046247

[B31] He X. Lillemo M. Shi J. Wu J. Bjørnstad Å. Belova T. . (2016). QTL characterization of Fusarium head blight resistance in CIMMYT bread wheat line Soru#1. PloS One 11, e0158052. doi: 10.1371/journal.pone.0158052 27351632 PMC4924825

[B32] Holland J. (2003). Estimating and interpreting heritability for plant breeding: An update. Available online at: https://www.scirp.org/reference/referencespapers?referenceid=1912064 (Accessed May 14, 2026).

[B33] Holzapfel J. Voss H.-H. Miedaner T. Korzun V. Häberle J. Schweizer G. . (2008). Inheritance of resistance to Fusarium head blight in three European winter wheat populations. Theor. Appl. Genet. 117, 1119–1128. doi: 10.1007/s00122-008-0850-z 18670751

[B34] Hu W. Gao D. Wu H. Liu J. Zhang C. Wang J. . (2020). Genome-wide association mapping revealed syntenic loci QFhb-4AL and QFhb-5DL for Fusarium head blight resistance in common wheat (Triticum aestivum L.). BMC Plant Biol. 20, 29. doi: 10.1186/s12870-019-2177-0 31959107 PMC6971946

[B35] Jeon D. Choi C. Park J. H. Kang C.-S. Kim J. Y. Kim C. (2026). Genomic selection for multi-trait crop improvement in wheat (Triticum aestivum L.): A practical modeling approach. BMC Plant Biol. 26, 400. doi: 10.1186/s12870-026-08258-7 41618159 PMC12933954

[B36] Jia G. Chen P. Qin G. Bai G. Wang X. Wang S. . (2005). QTLs for Fusarium head blight response in a wheat DH population of Wangshuibai/Alondra's. Euphytica 146, 183–191. doi: 10.1007/s10681-005-9001-7 30311153

[B37] Jin X. Jin X. Jia X. Wang H. Cao A. Zhao W. . (2013). Transcriptome-based discovery of pathways and genes related to resistance against Fusariumhead blight in wheat landrace Wangshuibai. Available online at: https://link.springer.com/article/10.1186/1471-2164-14-197 (Accessed April 1, 2026). 10.1186/1471-2164-14-197 PMC3616903 23514540

[B38] Jonas E. Fikse F. Rönnegård L. Mouresan E. F. (2019). “ Genomic selection,” in Population Genomics: Concepts, Approaches and Applications. Ed. Rajora O. P. ( Springer International Publishing, Cham), 427–480. doi: 10.1007/13836_2018_11

[B39] Jones S. P. T. (2015). The identification of physiological traits in wheat confering passive resistance to Fusarium head blight. Available online at: https://eprints.nottingham.ac.uk/28786/ (Accessed May 29, 2025).

[B40] Jones S. Farooqi A. Foulkes J. Sparkes D. L. Linforth R. Ray R. V. (2018). Canopy and ear traits associated with avoidance of Fusarium head blight in wheat. Front. Plant Sci. 9. doi: 10.3389/fpls.2018.01021 30108599 PMC6079624

[B41] Kubo K. Kawada N. Fujita M. (2013). Evaluation of Fusarium head blight resistance in wheat and the development of a new variety by integrating type I and II resistance. Japan Agric. Res. Quarterly: JARQ 47, 9–19. doi: 10.6090/jarq.47.9

[B42] Kumar S. Saharan M. S. Panwar V. Chatrath R. Singh G. P. (2019). Genetics of Fusarium head blight resistance in three wheat genotypes. Indian J. Genet. Plant Breed. 79, 614–617. doi: 10.31742/IJGPB.79.3.11

[B43] Kumar S. Stack R. W. Friesen T. L. Faris J. D. (2007). Identification of a novel Fusarium head blight resistance quantitative trait locus on chromosome 7A in tetraploid wheat. Phytopathology®. 97 (5). doi: 10.1094/PHYTO-97-5-0592 18943578

[B44] Li T. Bai G. Wu S. Gu S. (2011). Quantitative trait loci for resistance to fusarium head blight in a Chinese wheat landrace Haiyanzhong. Theor. Appl. Genet. 122, 1497–1502. doi: 10.1007/s00122-011-1549-0 21344182

[B45] Lin M. Islamov B. Aleliūnas A. Armonienė R. Gorash A. Meigas E. . (2024). Genome-wide association analysis identifies a consistent QTL for powdery mildew resistance on chromosome 3A in Nordic and Baltic spring wheat. Theor. Appl. Genet. 137, 25. doi: 10.1007/s00122-023-04529-1 38240841 PMC10799116

[B46] Lin F. Kong Z. X. Zhu H. L. Xue S. L. Wu J. Z. Tian D. G. . (2004). Mapping QTL associated with resistance to Fusarium head blight in the Nanda2419 × Wangshuibai population. I. Type II resistance. Theor. Appl. Genet. 109, 1504–1511. doi: 10.1007/s00122-004-1772-z 15290053

[B47] Lu Q. Morten L. Helge S. He X. Shi J. Fang J. . (2013). Anther extrusion and plant height are associated with Type I resistance to Fusarium head blight resistance in bread wheat line ‘Shanghai-3/Catbird. 126 (2), 126–317–334. doi: 10.1007/s00122-012-1981-9 23052019

[B48] Malihipour A. Gilbert J. Fedak G. Brûlé-Babel A. Cao W. (2017). Mapping the A genome for QTL conditioning resistance to Fusarium head blight in a wheat population with Triticum timopheevii background. Plant Dis. 101, 11–19. doi: 10.1094/PDIS-02-16-0144-RE 30682314

[B49] Mao S.-L. Wei Y.-M. Cao W. Lan X.-J. Yu M. Chen Z.-M. . (2010). Confirmation of the relationship between plant height and Fusarium head blight resistance in wheat (Triticum aestivum L.) by QTL meta-analysis. Euphytica 174, 343–356. doi: 10.1007/s10681-010-0128-9 30311153

[B50] Mardi M. Pazouki L. Delavar H. Kazemi M. B. Ghareyazie B. Steiner B. . (2006). QTL analysis of resistance to Fusarium head blight in wheat using a ‘Frontana’-derived population. Plant Breed. 125, 313–317. doi: 10.1111/j.1439-0523.2006.01228.x 40046247

[B51] Marroni F. Pinosio S. Zaina G. Fogolari F. Felice N. Cattonaro F. . (2011). Nucleotide diversity and linkage disequilibrium in Populus nigra cinnamyl alcohol dehydrogenase (CAD4) gene. Tree Genet. Genomes 7, 1011–1023. doi: 10.1007/s11295-011-0391-5 30311153

[B52] Mesterházy A. (1995). Types and components of resistance to Fusarium head blight of wheat. Plant Breed. 114, 377–386. doi: 10.1111/j.1439-0523.1995.tb00816.x 40046247

[B53] Mesterhazy A. (2024). What is Fusarium head blight (FHB) resistance and what are its food safety risks in wheat? Problems and solutions—a review. Toxins 16, 31. doi: 10.3390/toxins16010031 38251247 PMC10820574

[B54] Miedaner T. Reinbrecht C. Lauber U. Schollenberger M. Geiger H. H. (2001). Effects of genotype and genotype—environment interaction on deoxynivalenol accumulation and resistance to Fusarium head blight in rye, triticale, and wheat. Plant Breed. 120, 97–105. doi: 10.1046/j.1439-0523.2001.00580.x 37945311

[B55] Montesinos-López O. A. Chavira-Flores M. Kismiantini Crespo-Herrera L. Piere C. S. Li H. . (2024). A review of multimodal deep learning methods for genomic-enabled prediction in plant breeding. doi: 10.1093/genetics/iyae200 Available online at: https://academic.oup.com/genetics/article/228/4/iyae161/7876340 (Accessed April 22, 2026). PMC11631469 39499217

[B56] Morales L. Akdemir D. Girard A.-L. Neumayer A. Nannuru V. K. R. Shahinnia F. . (2024). Leveraging trait and QTL covariates to improve genomic prediction of resistance to Fusarium head blight in Central European winter wheat. Available online at: https://www.frontiersin.org/journals/plant-science/articles/10.3389/fpls.2024.1454473/full (Accessed April 22, 2026). 10.3389/fpls.2024.1454473 PMC11486744 39430891

[B57] Moreno-Amores J. Michel S. Miedaner T. Longin C. F. H. Buerstmayr H. (2020). Genomic predictions for Fusarium head blight resistance in a diverse durum wheat panel: an effective incorporation of plant height and heading date as covariates. Euphytica 216, 22. doi: 10.1007/s10681-019-2551-x 30311153

[B58] Muqaddasi Q. H. Reif J. C. Röder M. S. Basnet B. R. Dreisigacker S. (2019). Genetic mapping reveals large-effect QTL for anther extrusion in CIMMYT spring wheat. Agronomy 9, 407. doi: 10.3390/agronomy9070407 30654563

[B59] Nannuru V. K. R. Windju S. S. Belova T. Dieseth J. A. Alsheikh M. Dong Y. . (2022). Genetic architecture of fusarium head blight disease resistance and associated traits in Nordic spring wheat. Theor. Appl. Genet. 135, 2247–2263. doi: 10.1007/s00122-022-04109-9 35597885 PMC9271104

[B60] Okada T. Jayasinghe J. E. A. R. M. Eckermann P. Watson-Haigh N. S. Warner P. Hendrikse Y. . (2019). Effects of Rht-B1 and Ppd-D1 loci on pollinator traits in wheat. Theor. Appl. Genet. 132, 1965–1979. doi: 10.1007/s00122-019-03329-w 30899967

[B61] Paillard S. Schnurbusch T. Tiwari R. Messmer M. Winzeler M. Keller B. . (2004). QTL analysis of resistance to Fusarium head blight in Swiss winter wheat (Triticum aestivum L.). Theor. Appl. Genet. 109, 323–332. doi: 10.1007/s00122-004-1628-6 15014875

[B62] Pan Y. Liu Z. Rocheleau H. Fauteux F. Wang Y. McCartney C. . (2018). Transcriptome dynamics associated with resistance and susceptibility against fusarium head blight in four wheat genotypes. BMC Genomics 19, 642. doi: 10.1186/s12864-018-5012-3 30157778 PMC6116500

[B63] Parry D. W. Jenkinson P. McLeod L. (1995). Fusarium ear blight (scab) in small grain cereals—a review. Plant Pathol. 44, 207–238. doi: 10.1111/j.1365-3059.1995.tb02773.x 40046247

[B64] Pérez P. de los Campos G. (2014). Genome-wide regression and prediction with the BGLR statistical package. Genetics 198, 483–495. doi: 10.1534/genetics.114.164442 25009151 PMC4196607

[B65] Petersen S. Lyerly J. H. McKendry A. L. Islam M. S. Brown-Guedira G. Cowger C. . (2017a). Validation of Fusarium head blight resistance QTL in US winter wheat. Crop Sci. 57, 1–12. doi: 10.2135/cropsci2015.07.0415

[B66] Petersen S. Lyerly J. H. McKendry A. L. Islam M. S. Brown-Guedira G. Cowger C. . (2017b). Validation of Fusarium head blight resistance QTL in US winter wheat. Crop Sci. 57, 1–12. doi: 10.2135/cropsci2015.07.0415

[B67] Pugh G. W. Johann H. Dickson J. G. (1933). Factors affecting infection of Wheat heads by Gibberella saubiuetii. 46 (9), 771–797.

[B68] Reis E. M. Zoldan S. M. Zanata M. (2023). Interactions between temperature and wheat head wetting duration on fusarium head blight intensity. Summa Phytopathol. 49, e268908. doi: 10.1590/0100-5405/268908

[B69] Ren R. Foulkes J. Mayes S. Yang X. Ray R. V. (2016). Identification of novel quantitative trait loci for resistance to Fusarium seedling blight caused by Microdochium majus and M. nivale in wheat. Field Crops Res. 191, 1–12. doi: 10.1016/j.fcr.2016.03.011 38826717

[B70] Salman H. A. Kalakech A. Steiti A. (2024). Random Forest Algorithm Overview. Babylonian Journal of Machine Learning 2024, 69–79. doi: 10.58496/BJML/2024/007

[B71] Sari E. Berraies S. Knox R. E. Singh A. K. Ruan Y. Cuthbert R. D. . (2018). High density genetic mapping of Fusarium head blight resistance QTL in tetraploid wheat. PloS One 13, e0204362. doi: 10.1371/journal.pone.0204362 30307951 PMC6181299

[B72] Savary S. Willocquet L. Pethybridge S. J. Esker P. McRoberts N. Nelson A. (2019). The global burden of pathogens and pests on major food crops. Nat. Ecol. Evol. 3, 430–439. doi: 10.1038/s41559-018-0793-y 30718852

[B73] Schroeder H. W. Christensen J. J. (1963). Factors affecting resistance of wheat to scab caused by Gibberella zeae. Phytopathology 53, 831–838.

[B74] Shan G. (2022). Monte Carlo cross-validation for a study with binary outcome and limited sample size. BMC Med. Inform Decis Mak 22, 270. doi: 10.1186/s12911-022-02016-z 36253749 PMC9578204

[B75] Skinnes H. Semagn K. Tarkegne Y. Marøy A. G. Bjørnstad Å. (2010). The inheritance of anther extrusion in hexaploid wheat and its relationship to Fusarium head blight resistance and deoxynivalenol content. Plant Breed. 129, 149–155. doi: 10.1111/j.1439-0523.2009.01731.x 40046247

[B76] Skinnes H. Tarkegne Y. Dieseth J. Bjørnstad Å. (2008). Associations between anther extrusion and Fusarium head blight in European wheat. Cereal Res. Commun. 36, 223–231. doi: 10.1556/CRC.36.2008.Suppl.B.19

[B77] Srinivasachary S. Gosman N. Steed A. Hollins T. W. Bayles R. Jennings P. . (2009). Semi-dwarfing Rht-B1 and Rht-D1 loci of wheat differ significantly in their influence on resistance to Fusarium head blight. Theor. Appl. Genet. 118, 695–702. doi: 10.1007/s00122-008-0930-0 19034409

[B78] Srinivasachary Gosman N. Steed A. Simmonds J. Leverington-Waite M. Wang Y. . (2008). Susceptibility to Fusarium head blight is associated with the Rht-D1b semi-dwarfing allele in wheat. Theor. Appl. Genet. 116, 1145–1153. doi: 10.1007/s00122-008-0742-2 18347773

[B79] Steiner B. Buerstmayr M. Michel S. Schweiger W. Lemmens M. Buerstmayr H. (2017). Breeding strategies and advances in line selection for Fusarium head blight resistance in wheat. Trop. Plant Pathol. 42, 165–174. doi: 10.1007/s40858-017-0127-7 30311153

[B80] Syed S. Aleliūnas A. Armonienė R. Brazauskas G. Gorash A. (2025). GWAS analysis of Fusarium head blight resistance in a Nordic-Baltic spring wheat panel. Front. Plant Sci. 16. doi: 10.3389/fpls.2025.1604296 40772045 PMC12325435

[B81] Syed S. Aleliūnas A. Lillemo M. Gorash A. (2024). Analyses of wheat resistance to Fusarium head blight using different inoculation methods. Agronomy 14, 2415. doi: 10.3390/agronomy14102415 30654563

[B82] Syed S. Liatukas Ž. Gorash A. (2026). Role of morpho-phenological traits in passive resistance to Fusarium head blight in wheat. Agriculture 16, 188. doi: 10.3390/agriculture16020188 30654563

[B83] Szabó-Hevér Á. Lehoczki-Krsjak S. Tóth B. Purnhauser L. Buerstmayr H. Steiner B. . (2012). Identification and validation of fusarium head blight and Fusarium-damaged kernel QTL in a Frontana/Remus DH mapping population. Can. J. Plant Pathol. 34, 224–238. doi: 10.1080/07060661.2012.676571 37339054

[B84] Thakur A. Dhariwal R. Joshi A. K. Mishra V. K. Sharma S. Singh M. K. . (2025). Genome-wide association study for agronomic and yield-related traits in spring wheat (Triticum aestivum L.) germplasm. BMC Plant Biol. 25, 1499. doi: 10.1186/s12870-025-07263-6 41184756 PMC12581534

[B85] Verges V. L. Brown-Guedira G. L. Sanford D. A. V. (2021). Genome-wide association studies combined with genomic selection as a tool to increase Fusarium head blight resistance in wheat. Crop Breeding Genet. Genomics 3, e210007. doi: 10.20900/cbgg20210007

[B86] Wang J. Zhang Z. (2021). GAPIT version 3: Boosting power and accuracy for genomic association and prediction. Genomics Proteomics Bioinformatics Bioinf. Commons 19, 629–640. doi: 10.1016/j.gpb.2021.08.005 34492338 PMC9121400

[B87] Wang J. Zhang Z. (2026). GAPIT version 4: Integration of GWAS into genomic prediction. Mol. Biol. Evol. 43, 1–8. doi: 10.1093/molbev/msag107 42047095 PMC13183175

[B88] Wang D. Zhao Y. Zhao X. Ji M. Guo X. Tian J. . (2023). Genome-wide association analysis of type II resistance to Fusarium head blight in common wheat. PeerJ 11, e15906. doi: 10.7717/peerj.15906 37750077 PMC10518165

[B89] Wright M. N. Ziegler A. (2017). ranger: A fast implementation of random forests for high dimensional data in C++ and R. J. Stat. Software 77, 1–17. doi: 10.18637/jss.v077.i01

[B90] Würschum T. Jähne F. Phillips A. L. Langer S. M. Longin C. F. H. Tucker M. R. . (2020). Misexpression of a transcriptional repressor candidate provides a molecular mechanism for the suppression of awns by Tipped 1 in wheat. J. Exp. Bot. 71, 3428–3436. doi: 10.1093/jxb/eraa106 32103263 PMC7307850

[B91] Xu Y. Li Y. Bian R. Zhang G. Fritz A. K. Dong Y. . (2023). Genetic architecture of quantitative trait loci (QTL) for FHB resistance and agronomic traits in a hard winter wheat population. Crop J. 11, 1836–1845. doi: 10.1016/j.cj.2023.09.004 38826717

[B92] Xu Q. Xu F. Qin D. Li M. Fedak G. Cao W. . (2020). Molecular mapping of QTLs conferring Fusarium head blight resistance in Chinese wheat cultivar Jingzhou 66. Plants 9, 1021. doi: 10.3390/plants9081021 32806760 PMC7465298

[B93] Yannam V. R. R. Lopes M. S. Soriano J. M. (2025). Optimizing Genomic Selection Models for Wheat Breeding under Contrasting Water Regimes in a Mediterranean Environment. Plant Methods 21 (1), 155. doi: 10.1186/s13007-025-01467-5 41351168 PMC12679779

[B94] Yi X. Cheng J. Jiang Z. Hu W. Bie T. Gao D. . (2018). Genetic analysis of fusarium head blight resistance in CIMMYT bread wheat line C615 using traditional and conditional QTL mapping. Front. Plant Sci. 9. doi: 10.3389/fpls.2018.00573 29780395 PMC5946024

[B95] Yu J.-B. Bai G.-H. Zhou W.-C. Dong Y.-H. Kolb F. L. (2008). Quantitative trait loci for fusarium head blight resistance in a recombinant inbred population of Wangshuibai/Wheaton. Phytopathology® 98, 87–94. doi: 10.1094/PHYTO-98-1-0087 18943242

[B96] Zhang W. Francis T. Gao P. Boyle K. Jiang F. Eudes F. . (2018). Genetic characterization of type II fusarium head blight resistance derived from transgressive segregation in a cross between Eastern and Western Canadian spring wheat. Mol. Breed. 38, 13. doi: 10.1007/s11032-017-0761-2 30311153

[B97] Zhang Y. Yang Z. Ma H. Huang L. Ding F. Du Y. . (2021). Pyramiding of fusarium head blight resistance quantitative trait loci, Fhb1, Fhb4, and Fhb5, in modern Chinese wheat cultivars. Front. Plant Sci. 12, 694023. doi: 10.3389/fpls.2021.694023 34335661 PMC8317056

[B98] Zheng T. Hua C. Li L. Sun Z. Yuan M. Bai G. . (2021). Integration of meta-QTL discovery with omics: Towards a molecular breeding platform for improving wheat resistance to fusarium head blight. Crop J. 9, 739–749. doi: 10.1016/j.cj.2020.10.006 38826717

[B99] Zhou W. Kolb F. L. Yu J. Bai G. Boze L. K. Domier L. L. (2004). Molecular characterization of fusarium head blight resistance in Wangshuibai with simple sequence repeat and amplified fragment length polymorphism markers. Genome 47, 1137–1143. doi: 10.1139/g04-069 15644972

