# Correction: Enhancing genomic prediction of wheat resistance to Fusarium head blight through integration of passive resistance traits

**DOI:** 10.3389/fpls.2026.1945009

**Published:** 2026-07-30

**Authors:** Andrii Gorash, Shayan Syed, Gintaras Brazauskas

**Affiliations:** 1 Department of Cereal Breeding, Institute of Agriculture, Lithuanian Research Centre for Agriculture and Forestry, Akademija, Lithuania; 2 Laboratory of Genetics and Physiology, Institute of Agriculture, Lithuanian Research Centre for Agriculture and Forestry, Akademija, Lithuania

**Keywords:** fusarium head blight, genomic prediction, GWAS, spring wheat, wheat resistance to FHB

The title of this article was erroneously given as: “Enhancing genomic prediction of wheat resistance to fusarium head blight through integration of passive resistance traits”. The correct title of the article is “Enhancing genomic prediction of wheat resistance to Fusarium head blight through integration of passive resistance traits”.

Several numbers in the text were in error. A correction has been made to the section **Abstract**: “Comparing the genomic prediction performance on the entire set of markers with that based on set 1 from cross-validated GWAS and fixed covariates (days to heading and anther extrusion), an absolute increase in accuracy of 0.10 (0.60 vs. 0.70), and 0.10 (0.60 vs. 0.70) was observed using the RKHS, and rrBLUP models, respectively.”

The sentence has been corrected to: “Comparing the genomic prediction performance on the entire set of markers with that based on set 1 from cross-validated GWAS and fixed covariates (days to heading and anther extrusion), an absolute increase in accuracy of 0.10 (0.60 vs. 0.70), and 0.08 (0.62 vs. 0.70) was observed using the RKHS, and rrBLUP models, respectively.”

A correction has been made to the section 3 **Results**, 3.5.1 *Proportion of variance explained by separate marker sets in genomic prediction models for FHB severity*, Paragraph 1:

“Using set 1, the prediction accuracy was the highest in RKHS (r=0.79; R^2^ = 0.63) and close in rrBLUP (r=0.76; R^2^ = 0.62) and slightly less in RF (r=0.71; R^2^ = 0.50). Set 2 demonstrated lower predictive accuracy than set 1 (r=0.47, R^2^ = 0.22 for RKHS; r=0.46, R^2^ = 0.21 for rrBLUP; r=0.50, R^2^ = 0.25 for RF). The coefficient of determination (R^2^) indicated that set 1 explained 63%, 62%, and 50% of the phenotypic variation (FHB severity) in the RKHS, rrBLUP, and RF models, respectively. The second set of 87 SNP markers, which were significantly associated with morpho-phenological traits, alone explained 22%, 21%, and 25% of FHB severity in the RKHS, rrBLUP, and RF models, respectively. The RKHS and rrBLUP models outperformed the RF model in predictive accuracy when set 1 was included alone and when the two sets were combined. The accuracy was improved and the model explained greater phenotypic variation (2% of absolute increase), when two marker sets were combined in the RKHS model, while performance declined slightly in the rrBLUP and RF models, with absolute decreases of 2% and 4%, respectively (Figure 2).”

The paragraph has been corrected to: “Using set 1, the prediction accuracy was the highest in RKHS (*r* = 0.79; R^2^ = 0.63) and close in rrBLUP (*r* = 0.76; R^2^ = 0.62) and slightly less in RF (*r* = 0.72; R^2^ = 0.52). Set 2 demonstrated lower predictive accuracy than set 1 (*r* = 0.47, R^2^ = 0.22 for RKHS; *r* = 0.46, R^2^ = 0.21 for rrBLUP; *r* = 0.50, R^2^ = 0.25 for RF). The coefficient of determination (R^2^) indicated that set 1 explained 63%, 62%, and 52% of the phenotypic variation (FHB severity) in the RKHS, rrBLUP, and RF models, respectively. The second set of 87 SNP markers, which were significantly associated with morpho-phenological traits, alone explained 22%, 21%, and 25% of FHB severity in the RKHS, rrBLUP, and RF models, respectively. The RKHS and rrBLUP models outperformed the RF model in predictive accuracy when set 1 was included alone and when the two sets were combined. The accuracy was improved and the model explained greater phenotypic variation (2% of absolute increase), when two marker sets were combined in the RKHS model, while performance declined slightly in the rrBLUP and RF models, with absolute decreases of 1% and 7%, respectively (Figure 2).”

A correction has been made to the section 3 **Results**, 3.5.3 *Genomic prediction for FHB severity, based on the whole set of markers*, Paragraph 1: “The prediction accuracy, measured by Pearson’s correlation coefficient (r), was 0.60 for RKHS, 0.62 for rrBLUP, and 0.53 for RF, the corresponding R^2^ values were 0.36, 0.38 and 0.28, respectively (Figure 5).”

The sentence has been corrected to: “The prediction accuracy, measured by Pearson’s correlation coefficient (*r*), was 0.60 for RKHS, 0.62 for rrBLUP, and 0.53 for RF, the corresponding R^2^ values were 0.36, 0.39 and 0.28, respectively (Figure 5).”

A correction has been made to the section 4 **Discussion**, 4.3 *Genomic prediction for wheat resistance to FHB*, Paragraph 4: “In RF, including these traits as additional predictors increased R^2^ by 0.06 (6 percentage points).”

The sentence has been corrected to: “In RF, including these traits as additional predictors increased R^2^ by 0.04 (4 percentage points) (Figure 4).”

The original version of this article has been updated.

